# Sociodemographic characteristics of populations living near industrial land disposals of known and suspected carcinogens across the United States

**DOI:** 10.1038/s41370-026-00873-8

**Published:** 2026-04-23

**Authors:** Elizabeth Sharp, Caroline N. Pruitt, Jared A. Fisher, Abigail R. Flory, Barry I. Graubard, Mary H. Ward, Rena R. Jones, Jessica M. Madrigal

**Affiliations:** 1 Occupational and Environmental Epidemiology Branch, Division of Cancer Epidemiology and Genetics, National Cancer Institute, National Institutes of Health, Department of Health and Human Services, Rockville, MD USA; 2 Westat, Inc, Rockville, MD USA; 3 Biostatistics Branch, Division of Cancer Epidemiology and Genetics, National Cancer Institute, National Institutes of Health, Department of Health and Human Services, Rockville, MD USA; 4 School of Integrated Sciences, Sustainability, and Public Health, University of Illinois Springfield, Springfield, IL USA

**Keywords:** Landfills, Asbestos, Lead, cadmium, Polychlorinated biphenyls

## Abstract

**Background:**

Industrial facilities dispose of millions of pounds of chemical waste in U.S.-based landfills, surface impoundments, and underground injection wells annually, but information on populations living in proximity to these releases is limited.

**Objective:**

We quantified on-site land disposals of known and suspected human carcinogens and evaluated population characteristics associated with disposals in the 50 U.S. states and Puerto Rico.

**Methods:**

We linked land releases of known and suspected carcinogenic chemicals from regulatory data (2010–2018) to 2010 census tract data overall and by region. We estimated odds ratios and 95% confidence intervals for associations of population characteristics with land releases of arsenic, asbestos, benzene, beryllium, cadmium, formaldehyde, nickel, polychlorinated biphenyls (PCBs), cobalt, lead, and styrene, comparing tracts with the highest releases ( > median or 4^th^ quartile) to those with zero releases using population density-adjusted multinomial logistic regression models.

**Results:**

Known carcinogens with releases mostly to land included arsenic, asbestos, beryllium, cadmium, formaldehyde, nickel, and PCBs. Probable carcinogens included acrylamide, acrolein, cobalt, and hydrazine. Land disposal amounts were highest in the West and South. The tract proportion of Black population was associated with 8–31% higher odds of the highest land disposals of lead, cadmium, and formaldehyde. Increases in Hispanic and Latino population were associated with 29–33% higher odds of a tract having the highest disposals of benzene, asbestos, PCBs, and styrene. Measures of lower socioeconomic status were associated with the highest disposals, including 16–72% higher odds of tracts having the highest disposals per 10% increase in the proportion of families experiencing poverty.

**Significance:**

Our findings highlight differences between populations living in areas with industrial land disposals compared to areas without them. As most studies of industrial pollution focus on air exposure, this study illustrates the need to consider land disposals as potential sources of environmental exposure to known and suspected carcinogens.

**Impact:**

- This evaluation showed that millions of pounds of carcinogenic chemicals and metals from industrial sources are disposed of on land annually across the U.S. Over 70% of industrial releases of asbestos, formaldehyde, polychlorinated biphenyls, and metals like beryllium, cadmium, and nickel occur as on-site land disposals to landfills, surface impoundments, and underground wells, with most occurring in the Southern and Western U.S. The highest volume disposals of these chemicals and metals occurred in census tracts with higher proportions of population with lower levels of educational attainment and family income and higher levels of poverty and unemployment.

## Introduction

In the United States (U.S.) industrial facilities deposit millions of pounds of chemical and metal waste in landfills, surface impoundments, and underground injection wells annually [1]. Nearby populations may be exposed to chemicals and metals stored at these sites through multiple pathways including volatilization of chemicals to air, soil contamination, and leaching of chemicals into groundwater [2]. An additional concern for potential human exposure occurs during natural hazards such as flooding [3, 4], which could facilitate the transfer of contaminants from landfills or waste sites to nearby communities.

The facilities that most frequently deposit waste on-site include those in the metal mining, chemical manufacturing, hazardous waste management, electric utility, and primary metal manufacturing sectors [5]. Most studies of industrial pollution focus on air emissions [6–12] as a result, relatively little is known about the potential exposure to chemical and metal waste from on-site disposals among the U.S. population. The potential for human exposure from these disposals may vary by chemical, characteristics of the waste (e.g., treated or untreated) and of the disposal site, including type (e.g., landfill, surface impoundment), age, and operation. The few existing studies on this topic have been limited in focus, evaluating population patterns in the areas surrounding landfills. Although these studies have shown that hazardous waste landfills are disproportionately located in communities with large proportions of Black, Hispanic and Latino populations, and those of low socioeconomic status [13–15]. They have not described the specific pollutants disposed of at these sites. Further, to our knowledge, no study has quantified the amounts of known and suspected carcinogens disposed of at these U.S.-based sites, which may pose environmental and public health burden to the surrounding population.

In prior work, we evaluated industrial air emissions of organic and inorganic pollutants (i.e., chemicals and metals) classified as known and probable human carcinogens in U.S. census tracts, demonstrating unequal burden of several carcinogens among U.S. population groups that identified as Black, Hispanic and Latino, had low educational attainment, or were experiencing poverty [11, 12]. Here, we used regulatory data from the last decade to summarize on-site land disposals of known and probable carcinogens in landfills, surface impoundments, underground wells, and land treatment areas on a nationwide scale and extend our work to evaluate differences in population characteristics among census tracts with and without on-site disposals of these carcinogens across the U.S.

## Methods

### Releases of known and probable human carcinogens

Releases of chemicals and metals were based on 2010-2018 annual data recorded in the U.S. Environmental Protection Agency (EPA)’s Toxics Release Inventory (TRI) Basic Data Files (downloaded March 2022) [16]. The TRI program was established in 1986 and requires industrial facilities that employ 10 or more full-time employees and manufacture, process, or otherwise use specific chemicals and metals in quantities above threshold levels within in a given year to report their releases to EPA and to other designated State or Tribal officials [17]. Twenty-one chemicals and metals classified as known human carcinogens (group 1) and 30 classified as probable human carcinogens (group 2 A) by the International Agency for Research on Cancer (IARC) [18]. are tracked by the TRI program and had releases reported in 2010–2018 (Table 1). In our evaluation of the metals, we modeled sums of each metal and its respective compound. This included: arsenic and inorganic arsenic compounds; beryllium and beryllium compounds; cadmium and cadmium compounds; nickel (a possible human carcinogen; group 2B) and nickel compounds; cobalt and cobalt compounds (unclassified by IARC); and lead (a possible human carcinogen; group 2B) and inorganic lead compounds. We summarized on-site land releases during the period of 2010–2018 by type including disposals to landfills (in which wastes are buried), surface impoundments (uncovered holding areas used to volatize and/or settle waste materials), underground injection wells (in which fluids are injected below the lowest underground source of drinking water), and land treatment/application farming (a management technique in which waste containing a TRI-listed chemical is applied to or incorporated into soil) [19]. For comparison to land disposal amounts, we summarized on-site releases to air, combining air releases reported from stacks (i.e., from a point source that occurs through confined air streams such as stacks or ducts) with fugitive releases (i.e., do not occur through a confined air stream such as from an equipment leak) for each chemical.

**Table 1:** Summary of on-site releases (2010–2018) of 51 chemicals and metals classified as known and suspected (probable) human carcinogens by the International Agency for Research on Cancer (IARC) as reported to air, landfills, surface impoundments, underground injection wells, and land treatment in the conterminous United States, Alaska, Hawaii, and Puerto Rico.

	Total emissions (pounds)^a^	Air (%)	Land disposalspounds^a,b^ (% total emissions)	Landfill (%)	Surface imp. (%)	UG well (%)	Land Tx (%)	Census tracts, n^c^	Pop., n^c^
**Known carcinogens**									
1,2-Dichloropropane	493,292	96.5	12,784 (2.6)	2.6	0	0	0	2	8144
1,3-Butadiene	12,426,382	98.6	164,604 (1.3)	0.1	0	1.2	0	5	23,474
4,4’-Methylenebis	16,857	99.1	0	0	0	0	0	0	0
4-Aminobiphenyl	544	76.5	128 (23.5)	0	0	23.5	0	1	2537
Arsenic^d^	1,830,984,380	0	976,509,502 (53.3)	1.7	51.5	0	0.1	186	800,433
Asbestos	180,184,455	0	180,160,009 (100)	100.0	0	0	0	22	86,216
Benzene	36,123,797	82.3	6,248,027 (17.3)	4.4	0	12.8	0.2	61	239,204
Benzidine	222	100.0	0	0	0	0	0	0	0
Beryllium^d^	4,568,220	0.9	4,147,481 (90.8)	58.1	31.5	0	1.2	78	325,048
β-Naphthylamine	123	100.0	0	0	0	0	0	0	0
Bis(chloromethyl)ether	6215	100.0	0	0	0	0	0	0	0
Cadmium^d^	38,859,140	0.3	34,704,686 (89.3)	51.9	34.6	2.9	0	52	210,921
Ethylene oxide	2,497,312	97.9	21,772 (0.9)	0.5	0	0.4	0	5	18,144
Formaldehyde	168,926,134	26.0	123,218,835 (72.9)	0.4	0	72.5	0	85	339,701
Lindane	346	100.0	0	0	0	0	0	0	0
Nickel^d^	214,838,490	1.8	194,249,699 (90.4)	37.4	50.7	1.7	0.6	406	1,742,321
o-Toluidine	48,272	90.9	2648 (5.5)	0	1.1	4.4	0	2	19,268
Pentachlorophenol	721,708	0.3	713,613 (98.9)	98.9	0	0	0	2	6326
Polychlorinated biphenyls	37,089,339	0	37,080,168 (100)	100.0	0	0	0	25	96,045
Trichloroethylene	20,554,890	96.7	224,224 (1.1)	1.1	0	0	0	7	23,459
Vinyl chloride	4,716,175	98.3	78,573 (1.7)	1.7	0	0	0	2	3787
**Probable carcinogens**					0		0		
1,1,1-Trichloroethane	940,874	51.3	272,969 (29.0)	27.1	0	1.9	0	5	17,830
1,2,3-Trichloropropane	87,973	25.6	55,582 (63.2)	63.2	0	0	0	2	6841
1,3-Propane sultone	915	100.0	0	0	0	0	0	0	0
2-Mercaptobenzothiazole	171,013	5.8	123,744 (72.4)	0	72.4	0	0	1	4368
Acrolein	7,389,605	36.5	4,689,288 (63.5)	0	0	63.5	0	3	9001
Acrylamide	47,377,284	0.4	47,192,497 (99.6)	0.1	0	99.5	0	6	24,363
Aniline	17,766,838	4.7	16,910,748 (95.2)	0	0	95.2	0	12	50,873
Cobalt^d^	38,032,577	1.2	33,152,777 (87.2)	44.1	40.3	1.9	0.9	192	799,933
Creosote	2,814,343	73.3	364,406 (12.9)	12.9	0	0	0	4	7695
Diazinon	171,118	1.1	169,141 (98.8)	98.8	0	0	0	4	13,391
Dichloromethane	28,924,087	97.3	746,757 (2.6)	0.5	0	2.1	0	14	57,016
Diethyl sulfate	108,793	44.3	60,595 (55.7)	55.7	0	0	0	1	2638
Dimethyl sulfate	7078	98.9	0	0	0	0	0	0	0
Dimethylcarbamyl chloride	131	100.0	0	0	0	0	0	0	0
Epichlorohydrin	1,535,362	60.8	36,375 (2.4)	0.4	0	2.0	0	5	21,704
Ethyl carbamate	200,241	1.1	198,040 (98.9)	98.9	0	0	0	1	2,488
Glycidol	681,511	61.3	0	0	0	0	0	0	0
Hydrazine	1,629,773	0.9	1,614,840 (99.1)	0	0	99.1	0	5	37,028
Lead^c^	6,403,878,782	0.1	1,686,523,641 (26.3)	3.5	22.8	0	0	1120	4,941,255
Malathion	96,432	11.5	85,267 (88.4)	88.4	0	0	0	1	4352
N,N-Dimethylformamide	6,960,721	28.3	4,960,640 (71.3)	0	0	71.2	0	5	23,714
N-Nitroso-N-ethylurea	114	100.0	0	0	0	0	0	0	0
N-Nitroso-N-methylurea	74	100.0	0	0	0	0	0	0	0
ortho-Anisidine	2,720	78.8	0	0	0	0	0	0	0
Styrene^d^	241,650,978	97.3	6,444,506 (2.7)	1.8	0	0.9	0	29	128,404
Tetrabromobisphenol A	405,638	20.2	323,265 (79.7)	79.6	0	0.1	0	9	24,032
Tetrachloroethylene	7,916,879	93.8	468,544 (5.9)	5.1	0	0.8	0	17	66,197
Tetrafluoroethylene	1,938,737	86.8	0	0	0	0	0	0	0
Tris(2,3-dibromopropyl)phosphate	12	100.0	0	0	0	0	0	0	0
Vinyl fluoride	730,933	100.0	0	0	0	0	0	0	0

Surface imp: Surface impoundment; UG: underground injection well; Land tx: land treatment/application farming; Pop: population.

^a^ Pounds of annual releases self-reported by industrial facilities to the U.S. Environmental Protection Agency Toxics Release Inventory Program in 2010-2018 and summed in each tract during the 9-year period (for annual average pounds emitted, divide by 9).

^b^ Land disposals calculated as the total sum (pounds) of disposals to landfills, surface impoundments, underground wells, and land treatment.

^c^ Number of census tracts and total population within census tracts with land disposals of each agent.

^d^ Arsenic: arsenic and inorganic arsenic compounds; Beryllium: beryllium and beryllium compounds; Cadmium: cadmium and cadmium compounds; Nickel: nickel and nickel compounds; Cobalt: cobalt and cobalt compounds; Lead: lead and inorganic lead compounds; Styrene: styrene and styrene-7,8-oxide.

### Population characteristics

We excluded 243 tracts (0.3%) that had zero population in the 2010 census (e.g., airports) and obtained population data from the 2010 decennial U.S. census and the 2006–2010 American Community Survey to characterize area-level sociodemographic characteristics [20]. for the remaining tracts (*n* = 73,426) in the 50 U.S. states and Puerto Rico. Tract-level characteristics included the percentage of population by self-reported race and ethnicity, educational attainment less than high school, unemployment, renter-occupied housing, families below the federal poverty line, median family income, and the Yost index [21]. a composite of seven census-based socioeconomic factors including education, income, and unemployment. Within each of the eight race and ethnicity groups, we assessed the percentages of adults with less than a high school education and families below the poverty line. To evaluate the distribution of disposals by region, we grouped tracts by census division [22].

### Statistical analysis

We summed the land releases for each chemical and metal during the 2010–2018 period and describe these disposals overall and by census region for the 73,426 U.S. census tracts and the U.S. territories.

Given the low volume and/or isolated geography (i.e., few census tracts) of on-site disposals (pounds) for some chemicals, we focused our model-based analyses on the 11 chemicals and metals with land disposals in greater than 20 census tracts (arsenic, asbestos, benzene, beryllium, cadmium, formaldehyde, nickel, polychlorinated biphenyls (PCBs), cobalt, lead, and styrene). For these chemicals and metals, we conducted analyses of associations between tract-level population characteristics and land disposal amounts. Methods to create exposure metrics have been described [12]. Briefly, for each year from 2010 to 2018 we calculated the inverse distance-weighted (IDW) sum of the annual land releases within the tract for each chemical and metal, by weighting each facility’s releases by the linear distance between the facility and the population-based tract centroid. We categorized the averaged IDW sum of disposals for arsenic, cobalt, lead, and nickel (greater than 100 tracts with land disposals) into their respective metal-specific quartiles during the period of 2010–2018. We categorized asbestos, benzene, beryllium, cadmium, formaldehyde, PCBs, and styrene (greater than 20 but fewer than 100 tracts with disposals) by splitting the tracts with disposals into two groups based on the median disposal amount (less than/equal to or greater than the median).

We used population density-adjusted multinomial logistic regression models with robust variance estimation to estimate odds ratios (ORs) and 95% confidence intervals (CIs) comparing the highest category of disposals [greater than the median or quartile 4 (Q4)] to the referent group of zero disposals (dependent variable) for continuous population characteristics [(1%, American Indian/Alaska Native, Asian, multiracial, Native Hawaiian and Pacific Islander, and Other race population groups); (10%, Black, Hispanic, and White population groups); ($10,000, family income) or (10-unit, Yost index) increases]. The model equation followed this form for categories *k* = 1, 2, 3, 4 (cut points at the quartiles) and *k* = 1, 2 (cut point at the median):

$${{\mathrm{ln}}}\,\left(\frac{\Pr \left(Y=k\right)}{\Pr (Y=0)}\right)=\,{\beta }_{0k}+\,{\beta }_{1k\,}{Population}\,{Density}+\,{\beta }_{2k}\,{Population}\,{Characteristic}\,$$

Analyses were conducted using SAS version 9.4 and STATA/SE version 18.0.

## Results

**Fig. 1:**
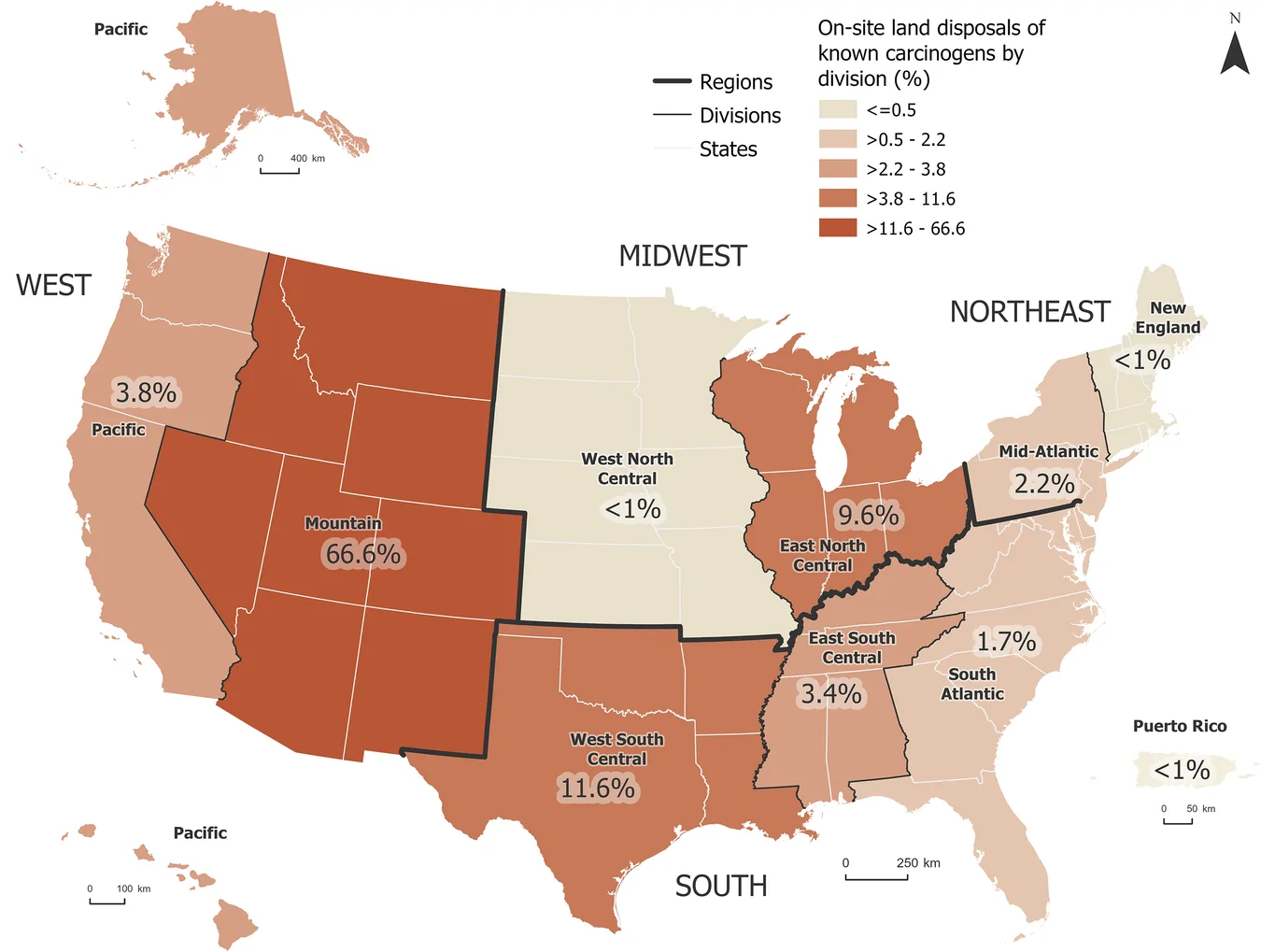
Distribution of reported on-site land disposals to landfills, surface impoundments, underground injection wells, and land treatment (2010–2018) of known carcinogens across the conterminous United States, Alaska, Hawaii, and Puerto Rico by census region and division. Land disposals are calculated as the total sum of disposals to landfills, surface impoundments, underground wells, and land treatment self-reported (in pounds) by industrial facilities to the U.S. Environmental Protection Agency Toxics Release Inventory Program in 2010-2018 and summed in each tract during the 9-year period. Census Division 1 (New England): Connecticut, Maine, Massachusetts, New Hampshire, Rhode Island, and Vermont; Division 2 (Mid-Atlantic): New Jersey, New York, and Pennsylvania; Division 3 (East North Central): Indiana, Illinois, Michigan, Ohio, and Wisconsin; Division 4 (West North Central): Iowa, Kansas, Minnesota, Missouri, Nebraska, North Dakota, and South Dakota; Division 5 (South Atlantic): Delaware, District of Columbia, Florida, Georgia, Maryland, North Carolina, South Carolina, Virginia, and West Virginia; Division 6 (East South Central): Alabama, Kentucky, Mississippi, and Tennessee; Division 7 (West South Central): Arkansas, Louisiana, Oklahoma, and Texas; Division 8 (Mountain): Arizona, Colorado, Idaho, New Mexico, Montana, Utah, Nevada, and Wyoming; Division 9 (Pacific): Alaska, California, Hawaii, Oregon, and Washington; As a U.S. territory, Puerto Rico is not classified under any census division or region.

**Fig. 2:**
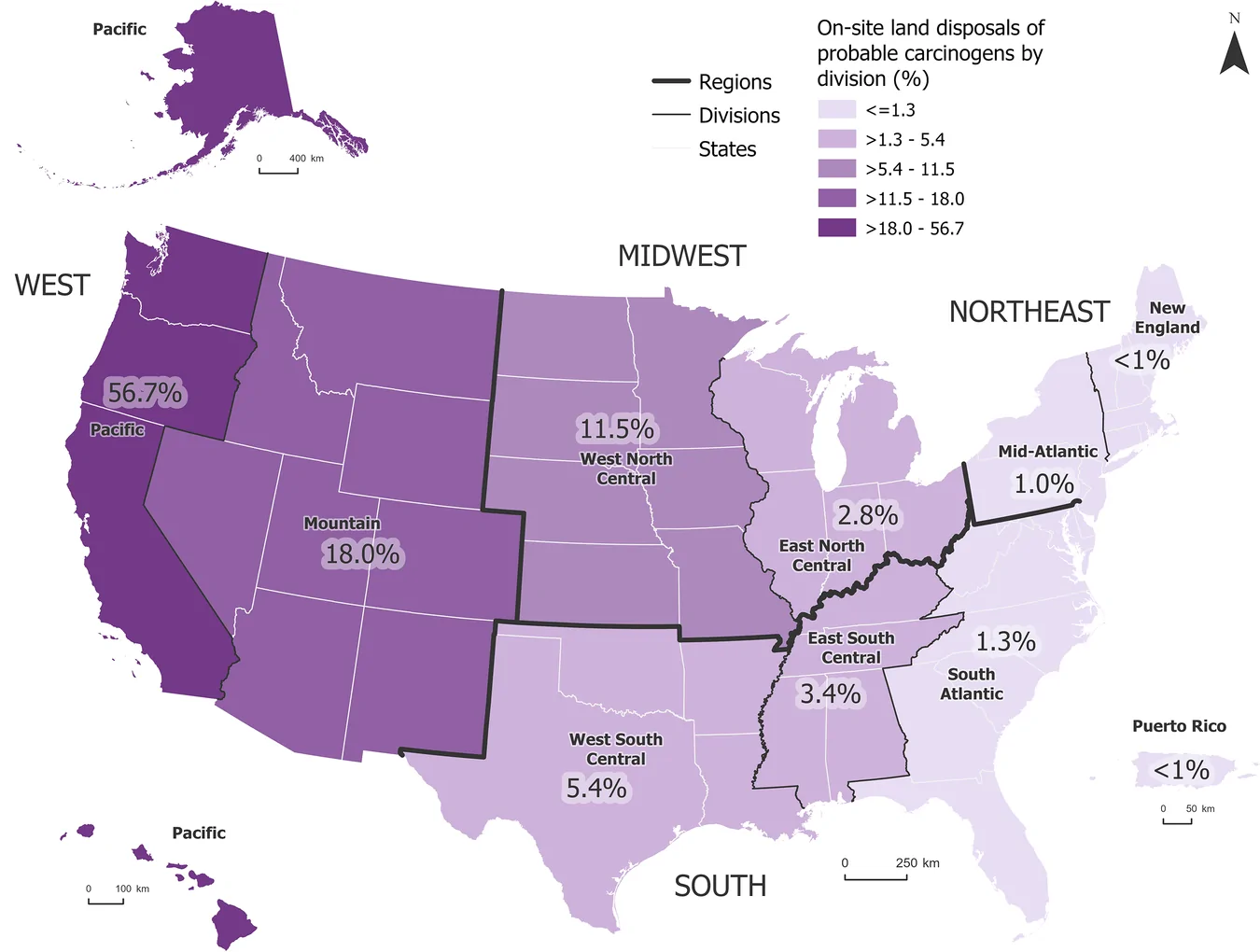
Distribution of reported on-site land disposals to landfills, surface impoundments, underground injection wells, and land treatment (2010–2018) of probable carcinogens across the conterminous United States, Alaska, Hawaii, and Puerto Rico by census region and division. Land disposals are calculated as the total sum of disposals to landfills, surface impoundments, underground wells, and land treatment self-reported (in pounds) by industrial facilities to the U.S. Environmental Protection Agency Toxics Release Inventory Program in 2010-2018 and summed in each tract during the 9-year period. Census Division 1 (New England): Connecticut, Maine, Massachusetts, New Hampshire, Rhode Island, and Vermont; Division 2 (Mid-Atlantic): New Jersey, New York, and Pennsylvania; Division 3 (East North Central): Indiana, Illinois, Michigan, Ohio, and Wisconsin; Division 4 (West North Central): Iowa, Kansas, Minnesota, Missouri, Nebraska, North Dakota, and South Dakota; Division 5 (South Atlantic): Delaware, District of Columbia, Florida, Georgia, Maryland, North Carolina, South Carolina, Virginia, and West Virginia; Division 6 (East South Central): Alabama, Kentucky, Mississippi, and Tennessee; Division 7 (West South Central): Arkansas, Louisiana, Oklahoma, and Texas; Division 8 (Mountain): Arizona, Colorado, Idaho, New Mexico, Montana, Utah, Nevada, and Wyoming; Division 9 (Pacific): Alaska, California, Hawaii, Oregon, and Washington; As a U.S. territory, Puerto Rico is not classified under any census division or region.

From 2010-2018, industrial facilities in the 50 U.S. states and Puerto Rico reported chemical and metal waste releases of known and suspected carcinogens on land in 1206 census tracts (1.6%) with an estimated population of 5.3 million people. Known human carcinogens with releases mostly to land (i.e., greater than 50% of releases) included arsenic, asbestos, beryllium, cadmium, formaldehyde, nickel, pentachlorophenol, and PCBs. Probable human carcinogens included acrylamide, acrolein, cobalt, and hydrazine. Although land disposals were reported for benzene (a known human carcinogen) and styrene (a probable human carcinogen) in 61 and 29 census tracts, respectively, over 80% of their releases were to air (Table 1). Some chemicals were rarely reported as land disposals (e.g., ethylene oxide: 0.9% of releases; trichloroethylene: 1.1% of releases) whereas others, including most of the metals, had a larger proportion (e.g., arsenic: 53.3%) or most of their disposals to land (e.g., asbestos, beryllium, cadmium, nickel, PCBs: 89.3% to 99.9%). For chemicals and metals with greater than 50% of releases to land, most were to landfills or surface impoundments, except for benzene, formaldehyde, acrolein, acrylamide, aniline, hydrazine, and N,N-dimethylformamide, for which the majority were deposited into underground wells. Lead, nickel, cobalt, and arsenic were the only agents disposed of to land in more than 100 census tracts. For both known and suspected carcinogens, land disposal amounts varied by census division and region with the highest overall proportions of combined disposals occurring in the Pacific and Mountain divisions in the West region (Supplementary Table 1). The West South Central and Mountain divisions in the South and West regions, respectively, had the largest proportions of known carcinogen disposals (Fig. 1; Supplementary Fig. 1), whereas the Pacific and Mountain divisions in the West region had the most disposals of probable carcinogens (Fig. 2; Supplementary Fig. 2).

In the U.S. territories, land disposals reported in the U.S. Virgin Islands included 18,241 pounds of nickel, 1546 pounds of cobalt, and 314 pounds of lead as land treatments and 22 pounds of lead in landfills. In Guam, 3000 pounds of lead were disposed of in landfills and 107 pounds in surface impoundments. In the Northern Mariana Islands, 6 pounds of lead were reported as released to surface impoundments. In Puerto Rico, 8,419,560 pounds of asbestos and 283,022 pounds of lead were disposed of in landfills; 70 pounds of lead were disposed of in surface impoundments. No land releases were reported in American Samoa.

**Fig. 3:**
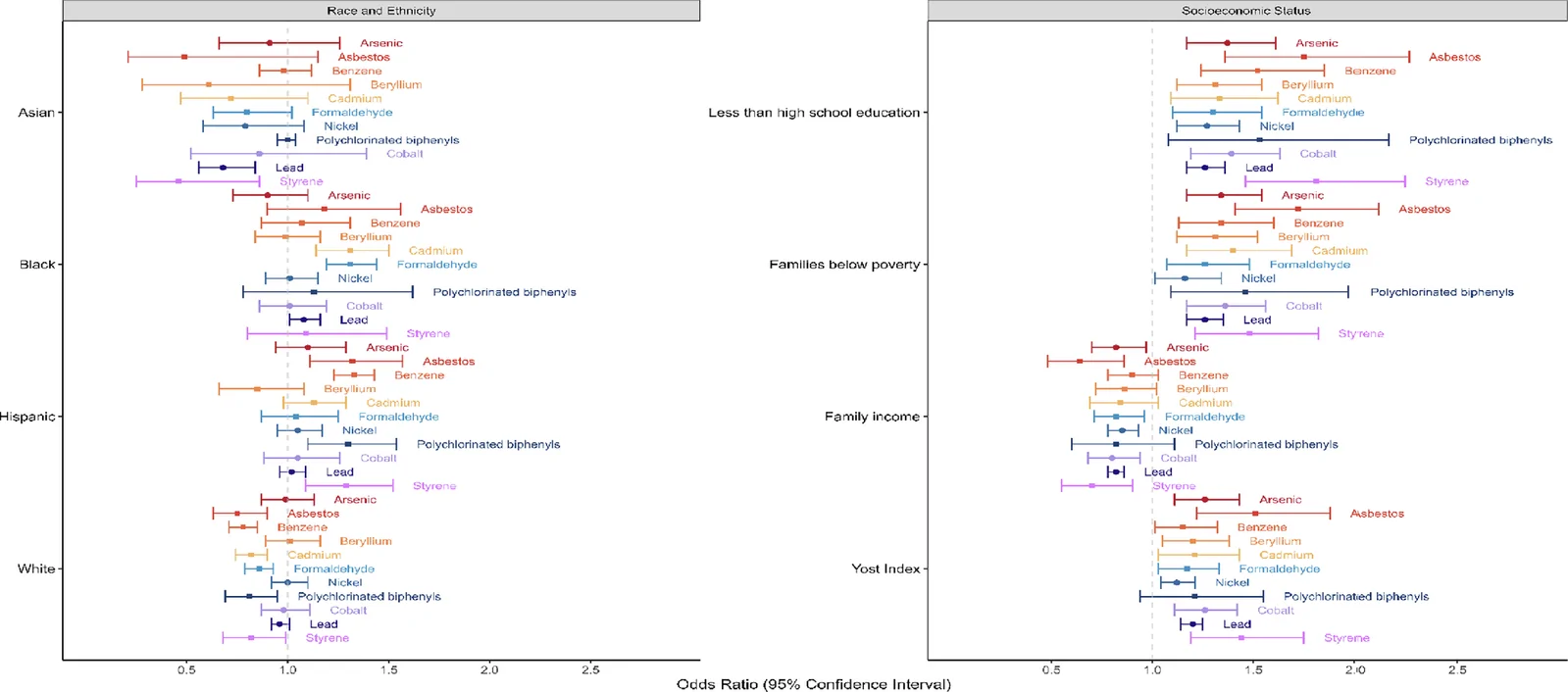
Associations^1^ of tract-level sociodemographic characteristics with odds of a tract having the highest^2^ agent-specific inverse-distance weighted (IDW) average land disposals within the tract (2010-2018). Left panel: Odds ratios and 95% confidence intervals population by race and ethnicity. Right panel: Odds ratios and 95% confidence intervals for population by measures of socioeconomic status. ^1^The reference category for each odds ratio is the group of census tracts with zero on-site land disposals of the chemical. Analyses restricted to agents with land disposals in a minimum of 20 census tracts; Models are adjusted for population density (population per km^2^ land area of the tract) and reflect a 10% increase in the characteristic of the tract population (i.e., a 10% increase in the percentage of the tract population with less than a high school education) except for Asian population where a 10% increase was not able to be estimated so a 1% increase in population characteristic was substituted; Median family income was missing for 493 tracts; Yost Index was missing for 1469 tracts ^2^For agents released in more than 100 tracts (arsenic, nickel, cobalt, and lead; estimate points presented with circle symbols) the distribution was categorized into quartiles, with estimates from a multinomial logistic model for the highest inverse-distance weighted (IDW) quartile (Q4) compared to zero disposals within the tract (non-exposed) presented; For agents with more than 20 but less than 100 exposed tracts (asbestos, benzene, beryllium, cadmium, formaldehyde, polychlorinated biphenyls, and styrene; estimate points are presented with square symbols) the IDW distribution was categorized into two categories split at the median (M).

Associations of population characteristics with land releases for eight known human carcinogens (arsenic, asbestos, benzene, beryllium, cadmium, formaldehyde, nickel, and PCBs) and three suspected carcinogens (cobalt, lead, and styrene). A 10% increase in the proportion of Black population in the census tract was associated with 8% to 31% greater odds of the tract having the highest land disposals (Q4 or greater than the median) of lead, cadmium, and formaldehyde compared to non-exposed (Fig. 3 left panel; Supplementary Tables 2, 3). Increases in Hispanic and Latino population were associated with 33%, 32%, 30%, and 29% higher odds of a tract having the highest disposals of benzene, asbestos, PCBs, and styrene, respectively. Conversely, increases in White population were not associated with higher odds of a tract having the highest disposals of any chemical or metal, and were associated with lower odds of the highest disposals of asbestos, benzene, cadmium, formaldehyde, PCBs, and styrene. Most of the indicators of low socioeconomic status were associated with the highest land disposals (Fig. 3 right panel; Supplementary Tables 2, 3). For the eleven chemicals and metals we evaluated, a 10% increase in the population proportion with less than a high school education was associated with 26% (lead) to 81% (styrene) higher odds of the tract having the highest disposals of these agents. Increases in the proportion of families experiencing poverty and in the Yost index of neighborhood deprivation were associated with 16–72% and 12–51% higher odds of tracts having the highest disposals of each chemical and metal, respectively. When we evaluated the joint associations of race and ethnicity with educational attainment and family poverty (Supplementary Tables 2, 3) odds remained elevated for beryllium, benzene, cadmium, and formaldehyde among Black populations with less than a high school education or experiencing poverty, and for all chemicals and metals except benzene and PCBs among White populations with less than a high school education or experiencing poverty.

## Discussion

This novel evaluation showed that there are millions of pounds of known and suspected carcinogenic chemicals and metals from industrial sources disposed of on land annually across the U.S. Over 70% of industrial releases of asbestos, formaldehyde, PCBs, and metals like beryllium, cadmium, lead, and nickel occur as on-site land disposals to landfills, surface impoundments, and underground wells, with most in areas within the Southern and Western U.S. For the eleven chemicals and metals we evaluated, most of which are known human carcinogens, we found that these disposals occurred most frequently in tracts with higher proportions of population with lower levels of educational attainment and family income and higher levels of poverty and unemployment, which is consistent with our prior studies of air emissions [11, 12]. Our land disposal findings add a chemical-specific nationwide evaluation to the prior literature showing that hazardous waste sites are generally located in areas where greater proportions of populations with low socioeconomic status live [13–15] by demonstrating that these associations persist when accounting for disposal amounts and for specific agents known or suspected to cause cancer in humans.

Health risks that have been associated with living in proximity to hazardous waste sites include adverse birth outcomes, asthma, and cancer [23, 24]. Exposure to the chemicals and metals we evaluated has been associated with lung, bladder, hematologic, ovarian, and other cancers [25–27]. with most of the epidemiologic studies evaluating risk from inhalation exposures. Relative to air and water releases, relatively little is known about the health effects of human exposure directly from land releases. Human exposure from waste disposal sites can occur through multiple exposure pathways including chemical and metal releases through volatile air emissions and contaminant migration into ground water, soil, or dust [2].

We found that most disposals of asbestos, beryllium, cadmium, and PCBs were to landfills; landfills at industrial sites are required to have a double liner and a double leachate collection and removal system as well as a leak detection system, controls for runoff, run-on, and wind dispersion, and a construction quality assurance program [28]. These measures are intended to limit the potential for human exposure to the contents of the landfill. However, degradation of the materials used to contain landfill waste over time, such as leachate collection and drainage systems [29]. geomembranes, drainage pipes [30]. and anti-seepage liner systems [31]. can shorten the service life of a landfill and increase the potential for leakage of high-concentration leachate [32]. Surface impoundments were the major disposal routes for arsenic and nickel; these sites also use double liner systems, leachate collection and removal systems, and leak detection systems as required by the Resource Conservation and Recovery Act (RCRA) regulations. Non-standardized operation and management practices during periods between mandated regulatory inspections have the potential to contribute to reduced performance and service lifespans of landfills and surface impoundments; for example, unit-specific inspection schedules for surface impoundments are set by the operator [33]. Formaldehyde and benzene, known human carcinogens, and a few suspected carcinogens (e.g., acrylamide, aniline, hydrazine, and styrene) were disposed of in underground injection wells, a method in which fluids are injected below the lowest underground source of drinking water [34]. Under the RCRA, compliance inspections by federal, state, local, and tribal program officials are required to be conducted on either an annual or biannual basis depending on facility ownership [35]. Despite the RCRA requirement, a 2022 report found that from 2015 through 2021, inspection compliance varied by type of site with only 91% of federal inspections and 63% of state, local, and tribal inspections completed [35]. Although regulations are in place for each of these disposal types to prevent human exposures, including the treatment of hazardous waste, exposure potential may vary by compound and by characteristics of the disposal site, including age, operation, and management practices.

In the absence of exposure studies, we cannot determine the human exposure from land releases at these sites. Few studies have evaluated associations between measurements of chemicals and metals present at actively managed industrial sites and concentrations from biological or household samples. Studies of Superfund sites, where hazardous waste has been dumped, left out in the open, or otherwise improperly managed, may provide insights regarding the exposure potential from land disposals of carcinogenic chemicals and metals. In an evaluation of exposure pathways at approximately 3000 hazardous waste sites from 1995 to 2004, the Agency for Toxic Substances and Disease Registry found that human exposure occurred at 30% of sites through more than one environmental medium (e.g., air, soil, water) and most commonly to metals (e.g., lead, arsenic, cadmium), volatile organic compounds, and PCBs [36]. Residential proximity to Superfund and other sites contaminated with metals and PCBs has been associated with concentrations of these compounds measured in toenails, serum, cord blood, and household dust [37–39]. Asbestos-contaminated waste sites are often capped with soil, however, at least one study has demonstrated the ability of asbestos fibers to travel through packed soil or sand, facilitated by dissolved organic matter [40]. These findings illustrate a possible human exposure pathway for asbestos fibers to move through the soil with the potential to leach into groundwater or become resuspended into the atmosphere [40]. Given that relatively small environmental concentrations of asbestos could have carcinogenic effects in human tissue [41]. This is one example of why monitoring the containment of these fibers at the industrial site to prevent human exposure could be critical. Mobility of other chemicals, such as heavy metals, through soil and groundwater as hazardous waste leachate is generally understudied and could vary widely depending on particle size, concentration, chemical species, and environmental factors, such as soil pH, type, and the presence of organic and inorganic matter [42–46]. For example, cadmium is most mobile and likely to leach into water under slightly acidic conditions [45], whereas lead is relatively immobile in soil, barring highly acidic situations [42].

This study has several strengths including the use of data for on-site disposals that spans nearly a decade for the entire U.S. and the U.S. territories. The census data used provides population characteristics from all racial and ethnic population groups in the 2010 decennial census. The metrics used for our analysis of associations incorporated distance- and population-weighting to provide a more detailed exposure characterization than the simpler metrics (e.g., counts of hazardous waste sites) that have been used in most other studies and better reflect the actual areas where the population resides and their exposure potential. However, we did not explore other distance weighting schemes, nor did we explicitly consider the influence of temperature, wind direction, chemical half-life, or other factors contributing to the fate and transport of any pollutant to reach the nearby population. Another limitation is the lack of information about human exposure. Living within a census tract that contains an industrial landfill, surface impoundment, or underground well serves as proxy for potential exposure and does not necessarily represent individual exposure to the agents in our study. Different types of disposals may yield different risks to the surrounding population; the potential for human exposure may vary widely and depend on the waste containment, treatment, and mitigation measures utilized at each individual site. We acknowledge the ecological design of this study and recognize that we have not evaluated local land use and zoning rules that govern the placement of industrial facilities permitted for on-site land disposals of carcinogenic chemical waste. Our analysis was not designed to determine whether certain population groups have moved into established industrial areas or if facilities that dispose of the carcinogens in our study are more likely to self-select to be sited within existing marginalized communities. The variability in tract size could have introduced spatial bias in our analysis. We attempted to avoid spatial bias to the extent possible by using the population center of the tract to derive the metrics we used in our analysis. Given the exploratory nature of this study, our interest is in evaluating the patterns of potential population exposure to these disposals. Therefore, we did not correct p-values for multiple testing. Instead, we interpreted our findings based on the magnitude of the estimates and consistency of the patterns of association observed. Our results need to be interpreted not only using the magnitude of the association and patterns, but also with the context of the data we have available. We used Census data for the population information; some population groups (e.g., Asian, Native Hawaiian and Other Pacific Islander) make up a small proportion of the U.S. population relative to other groups and therefore result in somewhat unstable estimates because our analysis aims to draw inferences about the U.S. on a nationwide scale. We interpret the associations for these groups with caution for that reason, not solely based on p-value.

## Conclusions

The on-site disposal of large quantities of known and probable human carcinogens across the Western and Southern U.S. highlights the need to conduct exposure studies to evaluate the potential for human exposure via air, soil, and water pathways among U.S. populations living near these industrial waste sites. Asbestos, formaldehyde, PCBs, and metals like arsenic, beryllium, cadmium, cobalt, lead, and nickel are disposed of at high volumes on land and, for some, in many census tracts across the nation. Presently, it is unclear if residential proximity to these disposals is associated with health risks. Our findings build on our prior evaluation of air emissions, highlighting the potential vulnerability of certain population groups who live near industrial sources that report high levels of on-site chemical and metal disposals.

## Supplementary information

## Data Availability

The data used in this study are publicly available from the U.S. EPA (https://www.epa.gov/toxics-release-inventory-tri-program) and the U.S. Census (https://www.nhgis.org/).
