# Supplementary material for: Sociodemographic characteristics of populations living near industrial land disposals of known and suspected carcinogens across the United States
Source: J Expo Sci Environ Epidemiol. 2026 Apr 23;36(5):910–8. doi: 10.1038/s41370-026-00873-8 (PMC13538043; doi:10.1038/s41370-026-00873-8)
Supplement: Supplementary file 1 [file 41370_2026_873_MOESM1_ESM.docx]

**Supplemental Materials**

**Sociodemographic characteristics of populations living near industrial land disposals of known and suspected carcinogens across the United States**

Elizabeth Sharp, Caroline N. Pruitt, Jared A. Fisher, Abigail R. Flory, Barry I. Graubard, Mary H. Ward, Rena R. Jones, Jessica M. Madrigal

Supplemental Table 1. Percentages (%) of census tracts with on-site land disposals**^a^** (2010-2018; includes disposals to landfills, surface impoundments, underground injection wells, and land treatment) of known and suspected (probable) human carcinogens distributed across the conterminous United States, Alaska, Hawaii, and Puerto Rico, by Census region and division^b^

|  |  | **Region 1: Northeast** | | **Region 2: Midwest** | | **Region 3: South** | | | **Region 4: West** | |  |
| --- | --- | --- | --- | --- | --- | --- | --- | --- | --- | --- | --- |
|  | **Census tracts, n^c^** | **New England** | **Mid-Atlantic** | **East North Central** | **West North Central** | **South Atlantic** | **East South Central** | **West South Central** | **Mountain** | **Pacific** | **Puerto Rico** |
| **Known carcinogens** |  |  |  |  |  |  |  |  |  |  |  |
| 1,2-Dichloropropane | 2 | 0 | 0 | 0 | 0 | 0 | 0 | 100 | 0 | 0 | 0 |
| 1,3-Butadiene | 5 | 0 | 0 | 0 | 0 | 0.02 | 0 | 92 | 8 | 0 | 0 |
| 4-Aminobiphenyl | 1 | 0 | 0 | 0 | 0 | 0 | 0 | 100 | 0 | 0 | 0 |
| Arsenic^d^ | 186 | <0.001 | 0 | 1 | 0.3 | 1 | 1 | 0.4 | 96 | 0.4 | 0 |
| Asbestos | 22 | 0 | 15 | 38 | 0 | 0 | 3 | 10 | 8 | 22 | 5 |
| Benzene | 61 | 0 | 0 | 0 | 30 | 0 | 0.2 | 45 | 0 | 25 | 0 |
| Beryllium^d^ | 78 | 0 | 1 | 30 | 0.1 | 16 | 15 | 10 | 19 | 10 | 0 |
| Cadmium^d^ | 52 | 0 | 0 | 4 | <0.001 | 4 | 41 | 25 | 24 | 2 | 0 |
| Ethylene oxide | 5 | 0 | 0 | 0 | 0 | 0 | 0 | 44 | 56 | 0 | 0 |
| Formaldehyde | 85 | <0.001 | 0 | 0.4 | 0.02 | 0.03 | 0.1 | 99 | 0 | <0.001 | 0 |
| Nickel^d^ | 406 | 0.01 | 2 | 29 | 2 | 8 | 8 | 12 | 34 | 5 | 0 |
| o-Toluidine | 2 | 0 | 0 | 0 | 0 | 0 | 0 | 100 | 0 | 0 | 0 |
| Pentachlorophenol | 2 | 0 | 0 | 30 | 0 | 0 | 0 | 0 | 0 | 70 | 0 |
| Polychlorinated biphenyls | 25 | 0 | 3 | 35 | 0 | <0.001 | 23 | 6 | 29 | 3 | 0 |
| Trichloroethylene | 7 | 0 | 0 | 1 | 0.3 | 1 | 0 | 0.1 | 5 | 92 | 0 |
| Vinyl chloride | 2 | 0 | 0 | 0 | 0 | 0 | 0 | 0.1 | 0 | 100 | 0 |
| **Probable carcinogens** |  |  |  |  |  |  |  |  |  |  |  |
| 1,1,1-Trichloroethane | 5 | 0 | 0 | 0 | 0 | 0 | 0 | 33 | 0 | 67 | 0 |
| 1,2,3-Trichloropropane | 2 | 0 | 0 | 0 | 0 | 0 | 0 | 100 | 0 | 0 | 0 |
| 2-Mercaptobenzothiazole | 1 | 0 | 0 | 0 | 0 | 0 | 0 | 0 | 100 | 0 | 0 |
| Acrolein | 3 | 0 | 0 | 0 | 0 | 0 | 0 | 100 | 0 | 0 | 0 |
| Acrylamide | 6 | 0 | 0 | 20 | 0 | 0 | 0 | 80 | 0.1 | 0 | 0 |
| Aniline | 12 | 0 | 0 | 0 | 0.001 | 0.01 | 0.001 | 100 | 0 | 0 | 0 |
| Cobalt^d^ | 192 | <0.001 | 2 | 20 | 3 | 15 | 11 | 13 | 33 | 4 | 0 |
| Creosote | 4 | 0 | 1 | 0 | 0 | 0 | 1 | 15 | 0 | 83 | 0 |
| Diazinon | 4 | 0 | 0 | 0 | 0 | 0 | 0 | 8 | 92 | 0 | 0 |
| Dichloromethane | 14 | 0 | 0 | 52 | 14 | 2 | 0 | 16 | 2 | 14 | 0 |
| Diethyl sulfate | 1 | 0 | 0 | 0 | 0 | 0 | 0 | 100 | 0 | 0 | 0 |
| Epichlorohydrin | 5 | 0 | 0 | 0 | 0 | 0 | 0 | 100 | 0 | 0 | 0 |
| Ethyl carbamate | 1 | 0 | 0 | 0 | 0 | 0 | 0 | 0 | 100 | 0 | 0 |
| Hydrazine | 5 | 0 | 0 | 1 | 0 | 0 | 0 | 99 | 0 | 0 | 0 |
| Lead^d^ | 1120 | 0.003 | 1 | 2 | 12 | 1 | 3 | 2 | 19 | 60 | 0.02 |
| Malathion | 1 | 0 | 0 | 0 | 0 | 0 | 0 | 0 | 100 | 0 | 0 |
| N,N-Dimethylformamide | 5 | 0 | 0 | 89 | 0 | 0 | 0 | 11 | 0 | 0 | 0 |
| Styrene^d^ | 29 | 0.03 | 0 | 1 | 0.004 | 0.1 | 1 | 47 | 0 | 51 | 0 |
| Tetrabromobisphenol A | 9 | 0 | 0 | 2 | 0 | 0 | 0 | 91 | 6 | 1 | 0 |
| Tetrachloroethylene | 17 | 0 | 0 | 22 | 3 | 0 | 0.1 | 0.3 | 3 | 71 | 0 |

^a^ Land disposals are calculated as the total sum of disposals to landfills, surface impoundments, underground wells, and land treatment self-reported (in pounds) by industrial facilities to the U.S. Environmental Protection Agency Toxics Release Inventory Program in 2010-2018 and summed in each tract during the 9-year period.

^b^ Division 1 (New England): Connecticut, Maine, Massachusetts, New Hampshire, Rhode Island, and Vermont; Division 2 (Mid-Atlantic): New Jersey, New York, and Pennsylvania; Division 3 (East North Central): Indiana, Illinois, Michigan, Ohio, and Wisconsin; Division 4 (West North Central): Iowa, Kansas, Minnesota, Missouri, Nebraska, North Dakota, and South Dakota; Division 5 (South Atlantic): Delaware, District of Columbia, Florida, Georgia, Maryland, North Carolina, South Carolina, Virgina, and West Virginia; Division 6 (East South Central): Alabama, Kentucky, Mississippi, and Tennessee; Division 7 (West South Central): Arkansas, Louisiana, Oklahoma, and Texas; Division 8 (Mountain): Arizona, Colorado, Idaho, New Mexico, Montana, Utah, Nevada, and Wyoming; Division 9 (Pacific): Alaska, California, Hawaii, Oregon, and Washington; As a U.S. territory, Puerto Rico is not classified under any census division or region.

^c^ Number of census tracts and total population within census tracts with land disposals of each agent;

^d^ Arsenic: arsenic and inorganic arsenic compounds; Beryllium: beryllium and beryllium compounds; Cadmium: cadmium and cadmium compounds; Nickel: nickel and nickel compounds; Cobalt: cobalt and cobalt compounds; Lead: lead and inorganic lead compounds; Styrene: styrene and styrene-7,8-oxide;

Supplemental Table 2. Associations of tract-level sociodemographic characteristics with odds of a tract having the highest^1^ agent-specific inverse-distance weighted (IDW) average land disposals within the tract (2010-2018) for known human carcinogens (Group 1).

| **Tract characteristics** | **Arsenic**  **OR_Q4_ (95% CI)^2^** | **Asbestos**  **OR_>M_ (95% CI)^2^** | **Benzene**  **OR_>M_ (95% CI)^2^** | **Beryllium**  **OR_>M_ (95% CI)^2^** | **Cadmium**  **OR_>M_ (95% CI)^2^** | **Formaldehyde**  **OR_>M_ (95% CI)^2^** | **Nickel**  **OR_Q4_ (95% CI)^2^** | **PCBs**  **OR_>M_ (95% CI)^2^** |
| --- | --- | --- | --- | --- | --- | --- | --- | --- |
| ***Race and ethnicity*** |  |  |  |  |  |  |  |  |
| **% AIAN**^3^ | 0.99(0.97-1.02) | 1.00(0.97-1.03) | 1.02(0.99-1.04) | 1.02(1.00-1.03) | 0.99(0.97-1.01) | 0.90(0.84-0.97) | 0.93(0.85-1.01) | 0.99(0.97-1.02) |
| **% Asian**^3^ | 0.91(0.66-1.26) | 0.49(0.21-1.15) | 0.98(0.86-1.12) | 0.61(0.28-1.31) | 0.72(0.47-1.10) | 0.80(0.63-1.02) | 0.79(0.58-1.08) | 1.00(0.95-1.04) |
| **% Black** | 0.90(0.73-1.10) | 1.18(0.90-1.56) | 1.07(0.87-1.31) | 0.99(0.84-1.16) | 1.31(1.14-1.50) | 1.31(1.19-1.44) | 1.01(0.89-1.15) | 1.13(0.78-1.62) |
| **% Hispanic** | 1.10(0.94-1.29) | 1.32(1.11-1.57) | 1.33(1.23-1.43) | 0.85(0.66-1.08) | 1.13(0.98-1.29) | 1.04(0.87-1.25) | 1.05(0.95-1.17) | 1.30(1.10-1.54) |
| **% Multiracial**^3^ | 0.98(0.88-1.09) | 0.60(0.25-1.45) | 0.91(0.74-1.12) | 1.00(0.94-1.07) | 1.00(0.93-1.07) | 0.91(0.78-1.06) | 0.87(0.76-1.00) | 0.97(0.82-1.14) |
| **% NHPI**^3^ | 0.96(0.73-1.26) | 0.93(0.50-1.76) | 0.82(0.32-2.13) | - | 1.02(0.96-1.08) | 0.75(0.17-3.28) | 0.94(0.78-1.15) | 1.02(0.97-1.06) |
| **% Other**^3^ | 0.96(0.46-2.00) | - | 1.10(0.97-1.26) | - | - | - | 0.27(0.04-1.87) | - |
| **% White** | 0.99(0.87-1.13) | 0.75(0.63-0.90) | 0.78(0.71-0.85) | 1.01(0.89-1.16) | 0.82(0.74-0.90) | 0.86(0.79-0.93) | 1.00(0.92-1.10) | 0.81(0.69-0.95) |
| ***Socioeconomic status*** |  |  |  |  |  |  |  |  |
| **% Less than HS education** | 1.37(1.17-1.61) | 1.75(1.36-2.26) | 1.52(1.24-1.85) | 1.31(1.12-1.54) | 1.33(1.09-1.62) | 1.30(1.10-1.54) | 1.27(1.12-1.43) | 1.53(1.08-2.16) |
| **% Rental housing^4^** | 1.24(1.09-1.40) | 1.05(0.76-1.44) | 1.16(0.99-1.35) | 1.06(0.88-1.27) | 1.25(1.06-1.47) | 1.05(0.88-1.25) | 1.14(0.99-1.30) | 1.42(1.09-1.85) |
| **% Unemployed** | 1.30(1.01-1.68) | 1.73(1.38-2.15) | 1.19(0.78-1.83) | 1.42(1.14-1.75) | 1.54(1.24-1.91) | 1.06(0.68-1.65) | 1.25(1.01-1.54) | 1.33(0.84-2.09) |
| **% Families <poverty** | 1.34(1.17-1.54) | 1.72(1.41-2.11) | 1.34(1.13-1.60) | 1.31(1.12-1.52) | 1.40(1.17-1.69) | 1.26(1.07-1.48) | 1.16(1.01-1.34) | 1.46(1.09-1.97) |
| **Family income ($10k)^5^** | 0.82(0.70-0.97) | 0.64(0.48-0.86) | 0.90(0.78-1.03) | 0.86(0.72-1.02) | 0.84(0.69-1.03) | 0.82(0.71-0.96) | 0.85(0.78-0.93) | 0.82(0.60-1.11) |
| **Yost Index (10 unit)^6^** | 1.26(1.11-1.43) | 1.51(1.22-1.88) | 1.15(1.01-1.32) | 1.20(1.05-1.38) | 1.21(1.03-1.43) | 1.17(1.03-1.33) | 1.12(1.04-1.21) | 1.21(0.94-1.55) |
| ***% Less than HS education*** | |  |  |  |  |  |  |  |
| **AIAN** | 1.00(0.91-1.11) | 1.05(0.83-1.32) | 1.05(0.93-1.19) | 0.87(0.74-1.03) | 0.99(0.87-1.13) | 0.90(0.74-1.09) | 0.93(0.83-1.03) | 0.93(0.74-1.17) |
| **Asian** | 0.74(0.57-0.95) | 1.00(0.79-1.28) | 0.99(0.88-1.13) | 0.70(0.47-1.02) | 0.89(0.69-1.16) | 1.03(0.91-1.18) | 0.98(0.88-1.08) | 1.05(0.88-1.25) |
| **Black** | 1.00(0.88-1.13) | 0.85(0.60-1.20) | 1.15(1.01-1.30) | 1.05(0.94-1.16) | 1.11(1.01-1.22) | 1.12(1.03-1.21) | 1.01(0.94-1.09) | 0.77(0.50-1.16) |
| **Hispanic** | 0.99(0.90-1.09) | 1.13(0.96-1.33) | 1.15(1.06-1.24) | 1.00(0.91-1.11) | 1.04(0.94-1.15) | 1.09(1.00-1.19) | 1.02(0.96-1.09) | 1.18(1.03-1.36) |
| **Multiracial** | 0.95(0.84-1.07) | 0.70(0.40-1.22) | 1.05(0.93-1.18) | 1.02(0.91-1.15) | 0.90(0.73-1.11) | 0.96(0.85-1.09) | 1.02(0.95-1.10) | 0.65(0.34-1.23) |
| **NHPI** | 1.16(0.99-1.35) | - | 1.06(0.85-1.32) | - | 1.13(0.91-1.40) | - | 1.09(0.96-1.25) | - |
| **Other** | 0.96(0.87-1.05) | 1.12(0.96-1.30) | 1.14(1.07-1.22) | 1.00(0.91-1.10) | 1.09(0.99-1.20) | 1.00(0.91-1.09) | 1.02(0.96-1.08) | 1.18(1.02-1.35) |
| **White** | 1.41(1.27-1.58) | 1.18(0.97-1.43) | 0.82(0.58-1.15) | 1.45(1.26-1.67) | 1.21(0.97-1.51) | 1.17(0.98-1.38) | 1.32(1.20-1.44) | 0.84(0.53-1.32) |
| ***% Families below poverty*** | |  |  |  |  |  |  |  |
| **AIAN** | 1.03(0.90-1.17) | 1.01(0.79-1.29) | 1.05(0.90-1.23) | 0.99(0.88-1.12) | 0.98(0.83-1.15) | 0.89(0.65-1.21) | 0.87(0.73-1.03) | 0.97(0.74-1.28) |
| **Asian** | 1.10(0.98-1.23) | 1.13(0.89-1.43) | 0.98(0.85-1.13) | 0.85(0.65-1.11) | 1.07(0.90-1.28) | - | 0.99(0.88-1.12) | - |
| **Black** | 1.05(0.94-1.17) | 1.05(0.90-1.23) | 1.10(0.97-1.25) | 1.14(1.04-1.26) | 1.10(0.99-1.21) | 1.11(1.04-1.19) | 1.02(0.94-1.11) | 0.95(0.74-1.21) |
| **Hispanic** | 1.03(0.93-1.14) | 1.03(0.85-1.26) | 1.04(0.95-1.13) | 0.98(0.87-1.10) | 0.99(0.86-1.15) | 1.01(0.90-1.13) | 0.95(0.87-1.04) | 1.01(0.90-1.14) |
| **Multiracial** | 0.99(0.87-1.13) | 1.06(0.86-1.30) | 1.08(0.97-1.22) | 0.98(0.84-1.14) | 0.90(0.67-1.21) | 0.97(0.83-1.13) | 0.95(0.84-1.06) | 0.84(0.61-1.15) |
| **NHPI** | 1.18(0.96-1.45) | 1.37(1.11-1.70) | 1.24(1.01-1.52) | - | 1.26(1.03-1.55) | - | 1.17(1.01-1.36) | 1.36(1.11-1.68) |
| **Other** | 1.00(0.89-1.12) | 1.16(1.00-1.35) | 1.06(0.96-1.18) | 0.59(0.38-0.94) | 0.96(0.85-1.08) | 1.01(0.91-1.13) | 0.92(0.84-1.02) | 0.94(0.78-1.14) |
| **White** | 1.38(1.25-1.53) | 1.24(1.00-1.54) | 1.11(0.86-1.43) | 1.40(1.27-1.55) | 1.07(0.80-1.43) | 1.16(0.98-1.38) | 1.18(1.05-1.32) | 1.25(0.78-2.01) |

^1^Analyses restricted to agents with land disposals in a minimum of 20 census tracts; For agents released in more than 100 tracts the distribution was categorized into quartiles, with estimates from a multinomial logistic model for the highest inverse-distance weighted (IDW) quartile (Q4) compared to zero disposals within the tract (non-exposed) presented; For agents with more than 20 but less than 100 exposed tracts the IDW distribution was categorized into two categories split at the median (M); ^2^ OR = odds ratio; CI = confidence interval; Odds ratios are adjusted for population density (population per km^2^ land area of the tract) and reflect a 10 percent increase in the characteristic of the tract population (i.e., a 10 percent increase in the percentage of the tract population with less than a high school education) unless otherwise noted; “—” indicate OR not presented. ^3^OR for 1% increase in population characteristic substituted when 10% increase was not able to be estimated; ^4^Percentage of rented housing units missing for 241 tracts; ^5^Median family income missing for 493 tracts; ^6^Yost Index missing for 1469 tracts; AIAN: American Indian and Alaska Native; NHPI: Native Hawaiian and Other Pacific Islander; HS: high school; PCBs: polychlorinated biphenyls;

Supplemental Table 3. Associations of tract-level sociodemographic characteristics with odds of a tract having the highest^1^ agent-specific inverse-distance weighted (IDW) average land disposals within the tract (2010-2018) for probable human carcinogens (Group 2A).

| **Tract characteristics** | **Cobalt**  **OR_Q4_ (95% CI)^2^** | | | **Lead**  **OR_Q4_ (95% CI)^2^** | | **Styrene**  **OR_>M_ (95% CI)^2^** |
| --- | --- | --- | --- | --- | --- | --- |
| ***Race and ethnicity*** |  | | |  | |  |
| **% AIAN**^3^ | 1.00(0.97-1.03) | | | 1.01(0.99-1.02) | | 0.88(0.75-1.03) |
| **% Asian**^3^ | 0.86(0.52-1.39) | | | 0.68(0.56-0.84) | | 0.46(0.25-0.86) |
| **% Black** | 1.01(0.86-1.19) | | | 1.08(1.01-1.16) | | 1.09(0.80-1.49) |
| **% Hispanic** | 1.05(0.88-1.26) | | | 1.02(0.96-1.09) | | 1.29(1.09-1.52) |
| **% Multiracial**^3^ | 0.96(0.87-1.06) | | | 0.92(0.84-1.02) | | 0.76(0.48-1.23) |
| **% NHPI**^3^ | - | | | 0.87(0.65-1.16) | | 1.02(0.97-1.08) |
| **% Other**^3^ | 1.01(0.78-1.30) | | | 0.34(0.10-1.14) | | - |
| **% White** | 0.98(0.87-1.11) | | | 0.96(0.92-1.01) | | 0.82(0.68-0.99) |
| ***Socioeconomic status*** |  | | |  | |  |
| **% Less than HS education** | 1.39(1.19-1.63) | | | 1.26(1.17-1.36) | | 1.81(1.46-2.24) |
| **% Rental housing^4^** | 1.20(1.01-1.42) | | | 1.10(1.01-1.19) | | 1.33(1.16-1.52) |
| **% Unemployed** | 1.43(1.20-1.71) | | | 1.27(1.09-1.48) | | 1.50(1.16-1.93) |
| **% Families <poverty** | 1.36(1.17-1.56) | | | 1.26(1.17-1.35) | | 1.48(1.21-1.82) |
| **Family income ($10k)^5^** | 0.80(0.68-0.94) | | | 0.82(0.78-0.86) | | 0.70(0.55-0.90) |
| **Yost Index (10 unit)^6^** | 1.26(1.11-1.42) | | | 1.20(1.14-1.25) | | 1.44(1.19-1.75) |
| **% Less than HS education** | |  |  | |  |  |
| **AIAN** | 0.99(0.88-1.11) | | | 0.99(0.95-1.04) | | 0.81(0.55-1.19) |
| **Asian** | 0.84(0.64-1.11) | | | 0.98(0.92-1.04) | | 0.85(0.61-1.20) |
| **Black** | 0.98(0.88-1.08) | | | 1.04(0.99-1.09) | | 1.06(0.89-1.27) |
| **Hispanic** | 1.05(0.96-1.15) | | | 1.04(1.00-1.08) | | 1.08(0.93-1.26) |
| **Multiracial** | 1.02(0.91-1.13) | | | 1.02(0.97-1.06) | | 0.88(0.70-1.10) |
| **NHPI** | 1.17(1.00-1.36) | | | 1.07(0.97-1.17) | | 1.15(0.93-1.43) |
| **Other** | 1.03(0.95-1.11) | | | 1.04(1.00-1.07) | | 1.06(0.93-1.22) |
| **White** | 1.47(1.33-1.62) | | | 1.25(1.18-1.33) | | 1.32(1.21-1.44) |
| **% Families below poverty** | |  |  | |  |  |
| **AIAN** | 0.95(0.82-1.09) | | | 1.02(0.96-1.07) | | - |
| **Asian** | 1.06(0.92-1.22) | | | 1.02(0.95-1.10) | | - |
| **Black** | 1.03(0.91-1.15) | | | 1.04(0.99-1.09) | | 1.16(0.99-1.36) |
| **Hispanic** | 0.97(0.86-1.09) | | | 1.01(0.97-1.06) | | 1.04(0.87-1.25) |
| **Multiracial** | 0.99(0.86-1.13) | | | 0.99(0.94-1.05) | | 0.89(0.73-1.09) |
| **NHPI** | - | | | 1.05(0.91-1.22) | | 1.34(1.09-1.65) |
| **Other** | 0.96(0.84-1.09) | | | 0.99(0.95-1.05) | | 1.01(0.90-1.13) |
| **White** | 1.38(1.25-1.51) | | | 1.20(1.13-1.27) | | 1.31(1.14-1.50) |

^1^Analyses restricted to agents with land disposals in a minimum of 20 census tracts; For agents released in more than 100 tracts the distribution was categorized into quartiles, with estimates from a multinomial logistic model for the highest inverse-distance weighted (IDW) quartile (Q4) compared to zero disposals within the tract (non-exposed) presented; For agents with more than 20 but less than 100 exposed tracts the IDW distribution was categorized into two categories split at the median (M); ^2^ OR = odds ratio; CI = confidence interval; Odds ratios are adjusted for population density (population per km^2^ land area of the tract) and reflect a 10 percent increase in the characteristic of the tract population (i.e., a 10 percent increase in the percentage of the tract population with less than a high school education) unless otherwise noted; “—” indicate OR not presented. ^3^OR for 1% increase in population characteristic substituted when 10% increase was not able to be estimated; ^4^Percentage of rented housing units missing for 241 tracts; ^5^Median family income missing for 493 tracts; ^6^Yost Index missing for 1469 tracts; AIAN: American Indian and Alaska Native; NHPI: Native Hawaiian and Other Pacific Islander; HS: high school;

Supplemental Figure 1. Locations of industrial facilities reporting on-site land disposals of known carcinogens to landfills, surface impoundments, underground injection wells, and land treatment across the conterminous United States, Alaska, Hawaii, and Puerto Rico by quintiles of average disposals (2010-2018) per census tract. Points represent census tract population-based centroids for each tract with at least one facility reporting on-site disposal.

Supplemental Figure 2. Locations of industrial facilities reporting on-site land disposals of probable carcinogens to landfills, surface impoundments, underground injection wells, and land treatment across the conterminous United States, Alaska, Hawaii, and Puerto Rico by quintiles of average disposals (2010-2018) per census tract. Points represent census tract population-based centroids for each tract with at least one facility reporting on-site disposal.
